# Transient Protection from Heat-Stress Induced Apoptotic Stimulation by Metastasis-Associated Protein 1 in Pachytene Spermatocytes

**DOI:** 10.1371/journal.pone.0026013

**Published:** 2011-10-12

**Authors:** Wei Li, Zhi-qun Wu, Jie Zhao, Sheng-jie Guo, Zhen Li, Xiao Feng, Li Ma, Jin-shan Zhang, Xin-ping Liu, Yuan-qiang Zhang

**Affiliations:** 1 Department of Human Anatomy, Histology and Embryology, Fourth Military Medical University, Xi'an, People's Republic of China; 2 Department of Interventional Radiology, Tangdu Hospital, Fourth Military Medical University, Xi'an, People's Republic of China; 3 Department of Urology, Cancer Center, Sun Yat-sen University, Guangzhou, People's Republic of China; 4 Department of Biochemistry and Molecular Biology, The State Key Laboratory of Cancer Biology, Fourth Military Medical University, Xi'an, People's Republic of China; University of South Florida College of Medicine, United States of America

## Abstract

**Background:**

Deregulated thermal factors have been frequently implicated in the pathogenesis of male infertility, but the molecular basis through which certain responses are directed remain largely unknown. We previously reported that overexpression of exogenous Metastasis-associated protein 1 (MTA1) protects spermatogenic tumor cells GC-2spd (ts) against heat-induced apoptosis. To further dissect the underlying mechanism, we addressed here the fine coordination between MTA1 and p53 in pachytene spermatocytes upon hyperthermal stimulation.

**Methodology/Principal Findings:**

High level of MTA1 expression sustained for 1.5 h in primary spermatocytes after heat stress before a notable decrease was detected conversely correlated to the gradual increase of acetylation status of p53 and of p21 level. Knockdown of the endogenous *MTA1* in GC-2spd (ts) elevated the acetylation of p53 by diminishing the recruitment of HDAC2 and thereafter led to a dramatic increase of apoptosis after heat treatment. Consistent with this, *in vivo* interference of MTA1 expression in the testes of C57BL/6 mice also urged an impairment of the differentiation of spermatocytes and a disruption of Sertoli cell function due to the elevated apoptotic rate after heat stress. Finally, attenuated expression of MTA1 of pachytene spermatocytes was observed in arrested testes (at the round spermatid level) of human varicocele patients.

**Conclusions:**

These data underscore a transient protective effect of this histone modifier in primary spermatocytes against heat-stress, which may operate as a negative coregulator of p53 in maintenance of apoptotic balance during early phase after hyperthermal stress.

## Introduction

About 88 years ago, Moore and his collaborators have provided the evidence for the first time that the scrotum is a local thermo-regulator and the temperature environment of the testes, which is below that of the general body temperature, is essential for the occurrence of normal spermatogenesis [1]. Retention of testes inside the abdomen, namely cryptorchidism, often observed in human birth defect regarding male genitalia, can result in disruption of spermatogenesis and impairment of steroidogenesis [2], [3]. Similarly in mice, precoital testicular heating not only reduced the number of successful matings, but also produced a transient retardation in embryo growth [4], [5]. Besides scrotum, a second thermoregulatory system is located in the spermatic cord where there is a counter-current heat exchange between incoming arterial blood and outgoing venous blood [6], [7]. The pathological dilation of testicular veins and pampiniform plexus, usually called varicocele, can elevate intrascrotal temperatures and thus is frequently associated with decreased conception rates among infertile couple [8], [9]. In short, accumulated studies have conclusively documented the adverse effects of hyperthermia on the normal adult testis among different species. Heat stress can result in disruption of the seminiferous epithelium, accumulation of lipid in Sertoli cells (Ser), local dilations of the intercellular spaces between Ser junctions, and increased apoptotic rate [10]. Based on histopathology, primary spermatocytes are the most susceptible cell type [11]. Although the physiological and cellular responses to heat treatment of the testes have been well documented, the molecular mechanisms through which these responses are directed remain largely unknown.

Metastasis-associated protein 1 (MTA1), a ubiquitously expressed chromatin modifier, plays an intergral role in nucleosome remodeling and histone deacetylase (NuRD) complexes [12], [13]. Soon after its identification, it became apparent that, in addition to its well-proven correlation with metastatic potential, MTA1 is also involved in the regulation of non-histone proteins by modifying the acetylation status of crucial target genes [14]. For example, during DNA double-strand break (DSB) repair in response to IR, MTA1 could directly stabilize the p53 protein by inhibiting its ubiquitination by E3 ligases, and therefore regulates p53-dependent function in DNA repair [15]. Interestingly, MTA1 could also inhibit p53-induced apoptosis by deacetylating p53, resulting in a more metastatic state in human cancer cells [16]. One possible explanation for these conflicting observations is that MTA1 may serve distinct roles in different physiopathological systems in response to different stimulations.

Our laboratory has a long-standing interest in the potential involvement of MTA1 during spermatogenesis. MTA1 protein is gradually increased in the testis since 14 days postnatal and reaches the maximum in adults, reversely correlating to the appearance of p53 in murine testis [17], [18]. Moreover, we have demonstrated that overexpression of MTA1 *in vitro* could remarkably elevate the capability of spermatogenic tumor cells against heat-induced apoptosis [19]. To better understanding the physiological function of MTA1 during normal spermatogenesis, we addressed here the distinctive interaction and regulatory roles between MTA1 and p53. Our data indicate that endogenous MTA1 can serve as a transient protector of primary spermatocytes against heat-stress induced apoptosis and therefore might act as a novel coregulator of p53 in maintenance of cellular integrity during early phase after hyperthermal stimulation in testicular germ cells.

## Results

### Tetraploid accumulation of MTA1 is not required for meiotic division but enhanced during acute phase of heat-stress induced apoptosis

**Figure 1:**
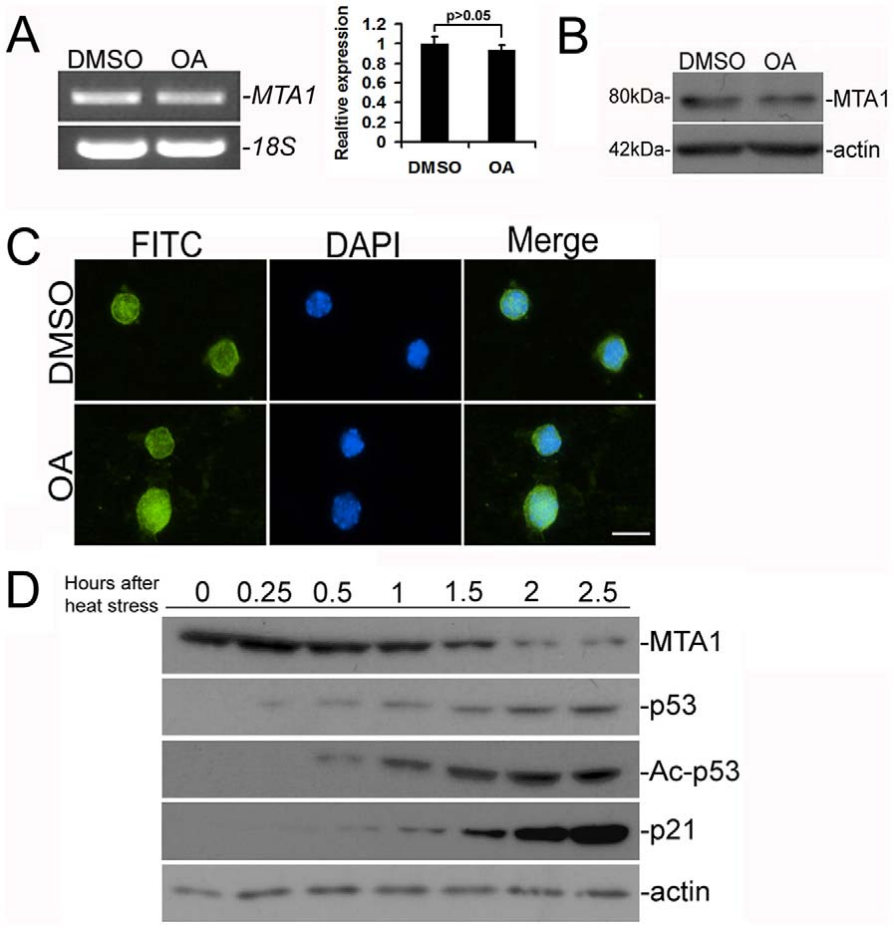
Expression of MTA1 was not required for meiotic division but was enhanced during acute phase of heat-stress induced apoptosis. **A** Expression of *MTA1* in isolated primary spermatocytes after OA treatment was examined using RT-PCR (left panel). PCR products were then quantified by SYBR green intercalation in real-time PCR (right panel). *18S* was served as an internal control for each PCR amplification. Data are expressed as mean±SEM (n = 3; p>0.05 vs. control). **B** Western analysis of MTA1 expression in isolated primary spermatocytes after OA treatment. The blot was reprobed with a β-actin (42 kDa) antibody to confirm equal loading. **C** Subcellular localization of MTA1 in isolated primary spermatocytes after OA treatment was determined by immunofluorescent staining. DAPI was used to stain nuclei of cells. *Bar = 10 µm* **D** Expression levels of MTA1, p53, Ac-p53 and p21 in primary spermatocytes were revealed at different time-points after heat stress by western blotting. β-actin was used as internal control.

MTA1 is mainly expressed in tetraploid primary spermatocytes and is slightly expressed in Ser of mouse testis, which suggests a potential involvement in meiosis [17]. To determine whether the predominant expression level of MTA1 is the prerequisite for the meiotic divisions of mouse spermatocytes, we treated isolated primary spermatocytes (Figure S1) in culture with okadaic acid (OA). This serine/threonine phosphatase inhibitor triggers entry of pachytene spermatocytes into meiotic divisions, which can be visualized by the overwhelming expression of phosphoH3 (Figure S2) [20]. Surprisingly, brief introduction of mid- to late pachytene spermatocytes to proceed to metaphase I was unable to stimulate the expression level of MTA1 at either transcriptional or translational levels (Fig. 1A and 1B). Unchanged nuclear localization of MTA1 after OA treatment also excludes the potential of being a ribonucleoprotein (RNPs) to favor mRNA translation during meiosis (Fig. 1C) [21]. MTA1 has been shown able to regulate the p53-dependent transcription for supplying nucleotides to repair damaged DNA in response to IR [15]. This raises the possibility that MTA1 might also play as a DNA damage responsive protein in coordination with p53 under hyperthermal condition. To preliminarily explore this assumption, we incubated primary spermatocytes at 43°C to induce heat stress and then analyzed the expression levels of MTA1 and of p53. There was a mild increase of MTA1 expression 0.25 h right after heat stress. This upregulation sustained for about 1 h before a dramatic decrease was observed at 2 h after heat stress (Fig. 1D). We also confirmed this expression profile at the transcriptional level (Figure S3). Consistent with the previous reports [22], [23], p53 expression gradually increased upon heat stimulation (first appearance of p53 expression was detected 0.25 h after heat stress). Acetylated status and functionality of p53 were also elevated from 0.5 h after heat stress, as evidenced by the expression level of Ac-p53 and p21. These data collectively suggested that there is a reverse correlation between the expression level of MTA1 and the activity of p53 induced by acetylation modification in tetraploid primary spermatocytes during acute phase of heat stimulation.

### MTA1 protects spermatocytes from temperature induced apoptosis by upregulation of HDAC2-mediated decetylation of p53

**Figure 2:**
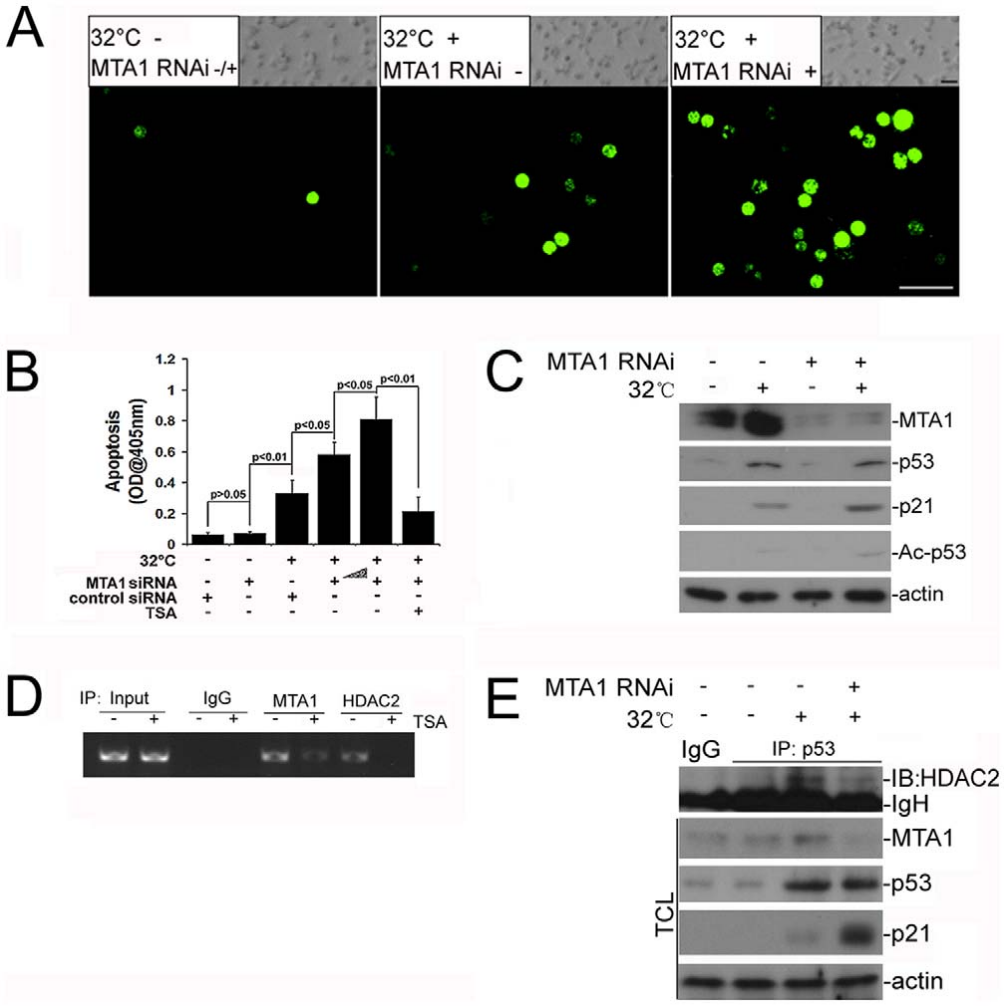
Impairment of the endogenous MTA1 in GC-2spd (ts) upregulated the acetylation of p53 by diminishing the recruitment of HDAC2 and led to an increase of apoptosis after temperature switch. **A** TUNEL staining of GC-2spd (ts) culture at 32°C for 6 h revealed a higher apoptotic wave due to the interference of MTA1 expression. Inserted phase images showed an equal cell density in two groups. *Bar = 20 µm* **B** Higher apoptotic rate in MTA1 RNAi GC-2spd (ts) was confirmed by ELISA methodology. **C** Inhibition of MTA1 expression only affected the deacetylation status and functionality of p53 instead of total p53 level. **D** Recruitment of *MTA1* and *HDAC2* to p53 promoter after heat stress was in a TSA-sensitive manner as shown by CHIP assay. **E** Endogenous association between p53 and HDAC2 induced by permission temperature (32°C) in GC-2spd (ts) was significantly decreased after suppression of MTA1 expression. Expression levels of Ac-p53 and p21 were also monitored in **D** and **E**.

Next, we try to figure out whether the endogenous MTA1 can perform certain roles in spermatogenic cells during apoptotic process induced by deregulated temperature. To address this question, we took advantage of a temperature sensitive spermatocyte-like cell line, namely GC-2spd (ts), which allows for full p53 nuclear localization at 32°C and partial p53 nuclear localization at 37°C but none at 39°C. The activity of the protein is determinant on the correct folding of p53, which is inhibited at the higher temperatures [24]. Moreover, these cells were derived from spermatocytes, the germ cell type very sensitive to heat-induced apoptosis [25]. Thus, GC-2spd (ts) affords experimentally an appropriately functional p53+/+ or p53 -/- *in vitro* model. After inhibiting about 78% expression level of *MTA1*/MTA1 (When compared to the internal control *18S*, the relative expression level of *MTA1* after RNAi was 0.2243±0.07, p = 0.0126) as demonstrated by real-time PCR and western blot analyses in Figure S4B, S4C, we subjected the GC-2spd (ts) into apoptotic permission temperature (32°C). The decrease of MTA1 expression notably increased the apoptotic susceptivity as histologically demonstrated by the elevated positive signals in TUNEL staining (Fig 2A). ELISA methodology also confirmed that the apoptotic rate of KD cells was significantly higher than that of control cells after heat stress (Fig. 2B). This apoptotic rate was trichostatin A (TSA)-sensitive and could be elevated when we achieved less expression of MTA1 by using higher level of siRNA (Fig. 2B, Figure S4D), confirming the indispensable role of MTA1 upon heat stress. Among the different candidates influencing the apoptotic process during spermatogenesis, p53 is believed to be a guardian of genome integrity [26]. MTA1 could inhibit p53-induced apoptosis by deacetylating p53 in cancer cells [16]. Therefore, we then focused our study on the potential relationship between these two molecules during heat stress induced apoptosis. As shown in Fig. 2C, the elevation of MTA1 expression induced by incubation at 32°C was not detected in knockdown (KD) groups. Surprisingly, inhibition of MTA1 expression could only affect the deacetylation status and functionality of p53 (as evidenced by the increasing expression level of Ac-p53 and of p21, respectively), instead of total p53 level. This indicated that endogenous MTA1 may exert effects on the activity of p53 at the post-translational level, probably through deacetylation modification. To further confirm that the *p53* gene chromatin is a direct target of MTA1, we performed ChIP assays in GC-2spd (ts) cultured at 32°C (Fig. 2D). Both *MTA1* and *HDAC2* could be recruited to p53 promoter in a TSA-sensitive manner, suggesting the possible involvement of nucleosome remodeling and histone deacetylase complex in the noted MTA1 regulation of *p53* transcription. After we knocked down the MTA1 expression, the endogenous association between p53 and HDAC2 induced by permission temperature was greatly reduced, reversely consistent with the evoked activity of p53 (as demonstrated by the increased expression level of p21) instead of total p53 level (Fig. 2E). Taken together, these findings identify MTA1 as a post-translational regulator of p53 through deacetylation modification upon heat stimulation.

### *In vivo* suppression of MTA1 increases heat-stress induced apoptotic rate and thereafter impairs the spermatogenic differentiation

**Figure 3:**
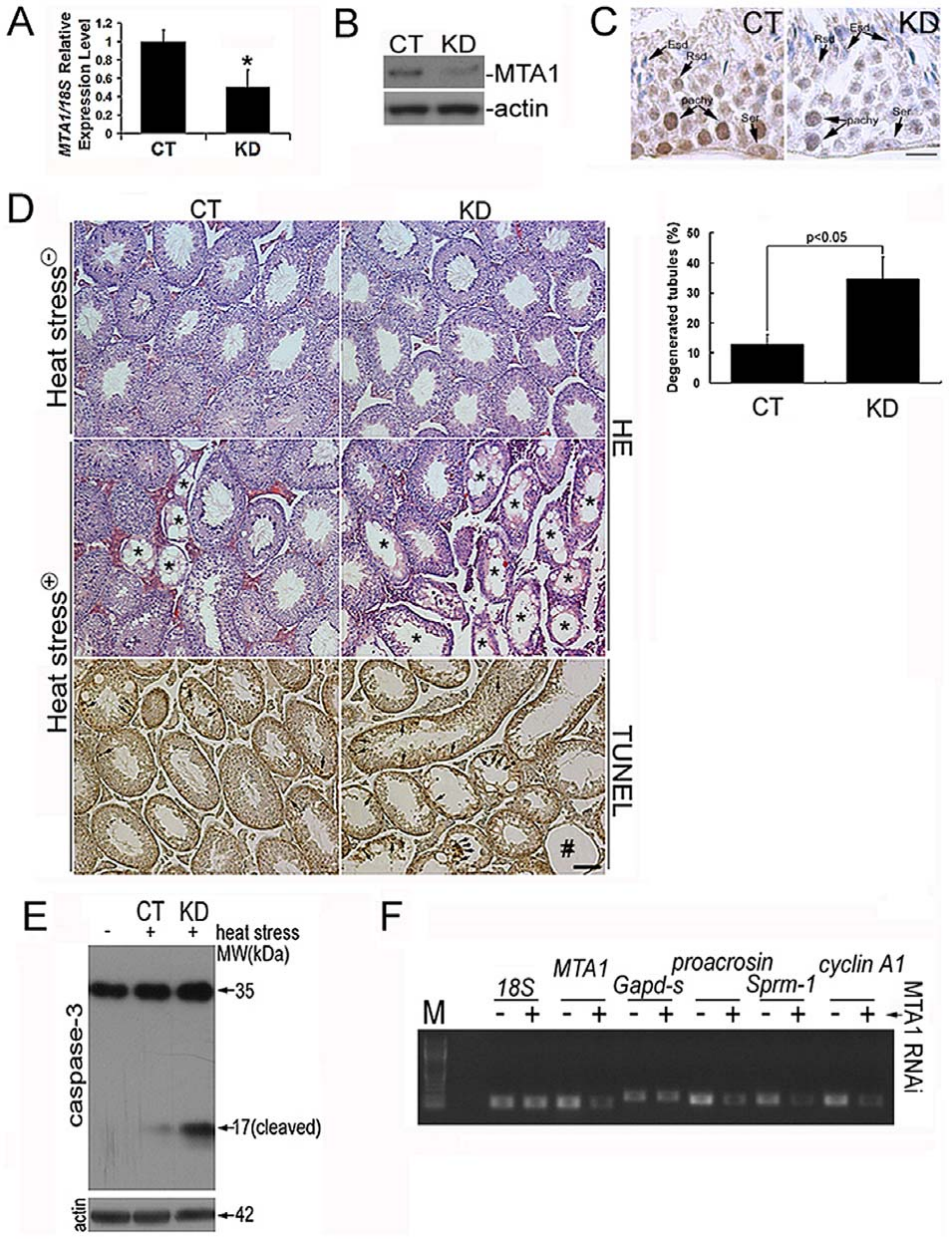
Increased apoptosis in a single, transient scrotal heat stress model after *in vivo* interference of MTA1 expression impaired the differentiation of primary spermatocytes. **A** Relative expression level of *MTA1/18S* was analyzed using real-time PCR 48 h after siRNA treatment. Data are expressed as mean±SEM (n = 3; ^*^p<0.05 vs. control). **B** Reduced expression of MTA1 was also confirmed by western blotting analysis. β-actin was used as internal control for equal loading. **C** Immunolocalization of MTA1 in mouse testicular sections treated with control siRNA and *MTA1* siRNA. Abbreviations: Ser, Sertoli cell; pachy, pachytene spermatocyte; Rsd, round spermatid; Esd, elongated spermatid. *Bar = 25 µm* **D** *In vivo* suppression of MTA1 had no effect on the histology of testis but resulted in more damaged seminiferous tubules and higher apoptosis 48 h after transient heat stress as shown by HE and TUNEL staining, respectively. Arrows indicated the apoptotic germ cells. *Bar = 50 µm* **E** Western blotting analysis of caspase-3 expression in testes treated with control siRNA and *MTA1* siRNA after transient heat stress. **F** The differentiating markers of primary spermatocytes not those of round spermatids were deregulated in *MTA1* siRNA-treated testis 48 after hyperthermal stimulation as demonstrated by RT-PCR. *18S* was served as an internal control.

The mammalian spermatogenesis carries out an amazing biological process. Significant knowledge about the molecular mechanisms of this process has been gained mainly by using genetically engineered animals, which however, are usually time-consuming and labor-intensive [27]. *In vivo* electroporation of the mammalian testis could reduce this burden because a gene of interest can be easily interrupted in target cells with adequate electric shock, and their behavior can be assayed directly [28]. To this end, we decided to use RNAi approach to knock down MTA1 gene expression *in vivo* to further elucidate its biological function. As shown in Fig. 3A and 3B, *MTA1* expression in the mouse testis was suppressed by 48.06±18.83% after 48 h by RNAi treatment as compared with the control group. This inhibition could be maintained until 72 h after treatment regardless of body metabolism (data not shown). Immunohistochemical examination revealed two cell types, namely pachytene spermatocytes (pachy) and Ser, which were deprived of MTA1 expression to the maximum extent (Figure 3C, Table 1, Figure S5). In line with the *in vitro* data, suppression of MTA1 expression failed to induce notable morphological difference under normal condition (Fig. 3D, HE staining) (To be noted, the tissue damages resulted from the injection of the plasmid and the electroporation manipulation have been taken into consideration when carrying out the histological examination). Instead, we found more degenerated seminiferous tubules (marked by asterisks in HE staining or by a pound key in TUNEL staining of Fig. 3D) in KD group 48 h after a single, transient scrotal heat stress stimulation. Further quantitative evaluation confirmed that the increased ratio of degenerated tubules in KD group was statistically significant (Fig. 3E). This degeneration was likely due to the upregulated apoptotic frequency as evidenced by TUNEL assay (Fig. 3D) and increased level of cleaved caspase-3 (Fig. 3F), respectively. Because the single testicular heating has been established as a reversible approach for impairment of spermatogenesis [29], we were then keen to examine whether this elevated apoptosis beard any biological effects. We explored the expression levels of genes known to be sequentially tuned in spermatocytes development. *Proacrosin* mRNAs are known to be expressed in mid-pachytene spermatocytes, and *Sprm-1* and *cyclin A1* mRNAs appear at the end of prophase of meiosis. Expression of the *Gapd-s* gene only begins in spermatids [30]. In accord with the deregulation of *MTA1* and the increased apoptotic rate in KD tubules, transcripts from the *Proacrosin*, *Sprm-1* and *cyclin A1* were practically decreased compared to the relatively steady level of *Gapd-s* mRNA (Fig. 3G). These data together suggested that the protective effect of MTA1 against heat stress induced apoptosis in primary spermatocyte was required for normal differentiation.

**Table 1:** Summary of the immunohistochemical analysis of MTA1 in mouse and human testes.

Group	Region/structure (Positive cell type)	Sub-cellular localization	Immunoreactivity	Positive cell number
CT mouse	Spermatogonium	Nuclei	-/2+ (moderate)	[2+] 25–50%
	Spermatocyte	Nuclei	3+ (strong)	4+ (>75%)
	Spermatid (round spermatid)	Nuclei	[-/+] faint	[-/+] <10%
	Spermatozoon	-	-	-
	Sertoli cell	Nuclei	1+ (weak)	[1+] 10–25%
	Leydig cell	-	-	-
KD Mouse	Spermatogonium	Nuclei	1+ (weak)	[-/+] <10%
	Spermatocyte	Nuclei	1+ (weak)	[3+] 50–75%
	Spermatid (round spermatid)	Nuclei	[-/+] faint	[-/+] <10%
	Spermatozoon	-	-	-
	Sertoli cell	Nuclei	[-/+] faint	[-/+] <10%
	Leydig cell	-	-	-
Nor Human	Spermatogonium	Nuclei	1+ (weak)	2+ (25–50%)
	Spermatocyte	Nuclei	3+ (strong)	3+ (50–75%)
	Spermatid (round spermatid)	Nuclei	3+ (strong)	4+ (>75%)
	Spermatozoon	-	-	-
	Sertoli cell	Nuclei	-/2+ (moderate)	1+ (10–25%)
	Leydig cell	-	-	-
Var Human	Spermatogonium	-	-	-
	Spermatocyte	-	-	-
	Spermatid (round spermatid)	Nuclei	2+ (moderate)	-/+ (<10%)
	Spermatozoon	-	-	-
	Sertoli cell	-	-	-
	Leydig cell	-	-	-

Score for positive cell number: [-/+] <10%, [1+] 10–25%, [2+] 25–50%, [3+] 50–75%, [4+] >75%.

Score for signal intensity: [-/+] faint, [1+] weak, [2+] moderate, [3+] strong.

### Impaired expression of MTA1 in hyperthermia related infertile testes

**Figure 4:**
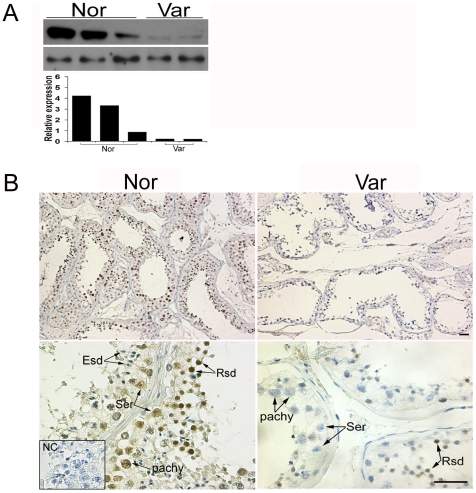
Attenuated expression of MTA1 in spermatogenesis-arrested testis (at the round spermatid level) of human varicocele patients. **A** Western analysis of MTA1 protein in normal (Nor) and varicocele (Var) testes. The blot was reprobed with β-actin antibody to confirm equal loading. **B** Immunohistochemical analysis of MTA1 expression in human pathological testes. Replacement of the primary antibody with normal goat IgG (NC) abolished the immunostaining in the tissues, confirming the specificity of the assay. *Bar = 20 µm*.

Varicocele is the pathological dilation of testicular veins and pampiniform plexus. The facts that varicoceles are found in a higher percentage among males attending the infertility clinics and that treatment of varicoceles is associated with increased spontaneous conception rates among infertile couples strongly suggest a deleterious effect of varicocele on male reproduction [31]. To date, the pathogenesis of varicocele is far from clear. However, emerging evidences have pointed out the elevated intrascrotal temperature due to retrograde flow in the affected veins as the top ranked aetiology involved [32], [33]. To better understand the pathological relevance of MTA1, therefore, we extended our investigation into the potential involvement of MTA1 in arrested spermatogenesis at the round spermatid level of varicocele. We observed a relatively lower level of MTA1 expression in pathological testis lysates (Fig. 4A). Subsequent immunohistochemical staining revealed a more detailed expression pattern. MTA1 beard the most intensive staining in the nuclear of round spermatid (Rsd) and of pachy in normal group, with a modest signal in the nuclear of some Ser. Weak staining was also observed in the nuclear of spermatogonia (spg). In contrast, elongate spermatids (Esd) and interstitial compartment did not exhibit any MTA1-specific immunostaining (Fig. 4B). Overall, the expression level of MTA1 in varicocele group (Var) was relatively lower at the low magnification. The moderate staining could only be detected in partial Rsd. Primary spermatocytes and Ser were deprived of positive signals. Replacement of the primary antibody with normal goat IgG (inserted window in Fig. 4B) abolished the immunostaining, confirming the specificity of immunohistochemical outcome. The details of immunohistochemical results were demonstrated in Table 1. Histochemical and biochemical assays have proved that MTA1 has a dominant expression in primary spermatocytes in both human and mouse testes [34], which led us to hypothesize that MTA1 might play a more conservative role in deciding the fate of tetraploid spermatocytes. To further verify the deteriorating effect of the impaired MTA1 expression on the status of primary spermatocytes, we went back to check the expression profile of MTA1 in the above-mentioned mouse transient scrotal heat stress model. An increase in TUNEL-positive apoptotic spermatocytes was not significantly obvious until 24 h after heat stress (Figure S7A). By contrast, the expression level of *MTA1* was decreased as early as 8 h after hyperthermal operation as compared to that of control group. In contrast to the *MTA1* expression, transient heat stress did not affect the expression of other genes, such as *vasa* and *pgk-2*, which are predominantly expressed in primary spermatocytes and/or round spermatids [35], [36], giving strong evidence that the reduction of *MTA1* was not due to a decrease in the number of cells (Figure S7B). As shown in Figure S7C, most primary spermatocyes at 8 h after heat stress, which had not yet undergone apoptosis as indicated by the normal morphology of chromatin, were deprived of the predominant nuclear staining of MTA1. These results suggested that the decrease of MTA1 expression level could be detected even earlier than the beginning of massive elimination of primary spermatocyes induced by hyperthermia.

## Discussion

Given the normal testicular function is temperature dependent, the testes are usually kept between 2 and 8°C below core body temperature by virtue of being held outside the body cavity in the scrotum [37]. However, the temperature of scrotum can be easily affected by numerous external factors such as posture, clothing, lifestyle, occupation and season [38], [39]. During the complicated process of spermatogenesis, the DNA of the spermatocytes in meiosis is most vulnerable to the introduction of a range of errors [40]. To this end, multifactorial protective mechanisms are warranted in spermatocytes to ensure the maintenance of cellular integrity upon stress conditions, such as DNA damage and hyperthermia. In fact, the expression of a number of DNA repair genes such *Ogg1* (involved in base excision repair), *Xpg* (involved in nucleotide excision repair) and *Rad54* (involved in double-strand break repair) are all down-regulated following heat stress at 43°C in murine testis [41]. Emerging literatures also establish MTA1 to be a valid DNA-damage responsive protein [42]. Brief induction of G2/M progression of primary spermatocytes by OA treatment could not upregulate the expression of MTA1, indicating that MTA1 is not necessarily tuned in the pathway of meiotic division triggered by OA. Actually, depletion of MTA1 can result in a defect of G2/M checkpoint in human cancer cells on the premise of UV radiation [43]. Thus, MTA1 may participate in checkpoint pathway under certain pathological conditions. In line with this assumption, our *in vitro* data showed an attenuated expression profile of MTA1 in primary spermatocytes within 2 h after hyperthermal stimulation, raising the possibility that it may help to maintain the optimum DNA-repair activity in germ cells exposed to heat stress during the acute phase.

In our previous study on the androgen-regulated expression of *MTA1* in mouse epididymis, we found an unexpectedly sharp increase of the *MTA1* mRNA level at the 5^th^ day of androgen supplementary treatment in the castrated animals, which was contrasted to the relatively lower circulated androgen level at this time-point [44]. This observation raised the possibility that *MTA1* expression was actually modulated by androgen signaling but it may also be transiently evoked by certain pathological conditions. After all, *MTA1* is a DNA-damage response gene and castration is an intrinsic stress condition. Similarly in the present study, heat stress could be another stimulator to the upregulation of MTA1 expression during the very early phase. This may explain why MTA1 expression was maintained at relatively high level from 0.25 h to 1 h after heat stress, regardless of a reverse correlation between the expression level of MTA1 and the activity of p53 induced by acetylation modification in tetraploid primary spermatocytes thereafter.

Next, we determined the biological effect of biochemical ablation of MTA1 in spermatocyte-like cells. Disruption of MTA1 expression could introduce remarkable increase of apoptotic rate in GC-2spd (ts) cultured at p53-active temperature. Interestingly, this endogenous inhibition did not impair the total level of p53, which was observed in our prior MTA1-overexpressing system [19]. Two possibilities were suggested for this discrepancy. Firstly, there are not necessarily relevant results capable of being obtained when introducing suppression of endogenous gene or overexpression of exogenous gene. For example, knockdown of candidate dyslexia susceptibility gene (CDSG) homologs in cerebral cortical progenitor cells results in acute disturbances of neocortical migration, while overexpression of CDSG does not have any effect [45]. Secondly, overexpression of exogenous MTA1 could induce at least 4-fold increase of target gene while in the *in vitro* knockdown GC-2spd (ts), we only detected about 50% reduction of MTA1 expression. It is possible that this overwhelming overexpression might activate other pathway to compromise the expression level of p53. These possibilities are being addressed in ongoing experiments.

**Figure 5:**
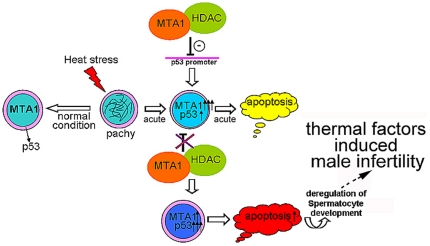
Summary diagram of the possible mechanisms related to MTA1 function contributing to transient protection of pachytene spermatocytes following heat stress.

Nevertheless, we did find the evidence that suppression of endogenous MTA1 in GC-2spd (ts) could reduce the recruitment of HDAC2 into p53 promoter, thus leading to a less deacetylated status of p53 and a parallel elevated acetylation of p53 (Fig. 2C, 2D). The acetylation of p53 is generally believed to be synonymous with the activation of the tumor suppressor, so we reasoned that the inhibition of MTA1 expression could result in the increased p53 activity upon heat stimulation, as evidenced by the increased expression level of p21 and thereafter a higher apoptotic rate in primary spermatocytes after heat stress. MTA1 may serve as a negative coregulator of p53 in spermatocytes. After heat shock, p53-mediated spontaneous testicular apoptosis is helpful to remove defective germ cells during initial phase and is subsequently followed by Fas-dependent apoptosis [23]. However, the p53-governed effect should not be exaggerated. For example, adenovirus-mediated p53 gene overexpression to rodent spermatocytes significantly impairs spermatogenesis [46]. Our data also demonstrated a deteriorating effect of this overdosed apoptotic wave on the spermatocyte differentiation after heat treatment. Given the facts that p53 is responsible for the initial phase of germ cell apoptosis induced by hyperthermia and MTA1 expression was also maintained at a relatively high level during acute phase in primary spermatocytes after heat stress, we hypothesize that MTA1 acts as a “buffer solution” to ensure the p53 activity below the threshold within the acute phase after hyperthermal stimulation. This apoptotic balance could be achieved by elevating the deacetylation level of p53 (Fig. 5).

As expected, inhibition of *in vivo* MTA1 expression induced a significant increase of apoptosis upon hyperthermal treatment at 48 h after 43°C hyperthermal stimulation. This elevated apoptotic rate finally resulted in an impairment of late development of spermatocytes. Meiosis in male mice occurs over a 2 week period [47]. Therefore, the relatively transitory experimental duration in our model (about 2 days after heat treatment) was not enough for any change of spermatids to occur. Besides primary spermatocytes, Ser was the other major cell type affected by the interference. This notion was supported by the observation that the expression of a number of genes essential for the complex interaction between germ cells and Ser was slightly disrupted in MTA1 KD testis after heat shock (Figure S6) [48]. A single exposure of the rodent testis to 43°C can only produce specific damage limited to the spermatocytes [49], [50]. Therefore, this disruption of Ser function is probably a subsequent result due to the apoptosis of germ cells. After all, Ser fate is also determined by the status of contacting germ cells [51]. Moreover, elevated temperature in adult mouse testis results in a complex stress response, including induction of genes associated with oxidative stress and hypoxia [20]. MTA1 exists in a biochemically distinct protein complex which comprises HDAC1/2, RbAp46/48, and MBD3 components. MTA1 induces the deacetylation of HIF-1alpha by increasing the expression of HDAC1 [42].In this context, MTA1 also bear potential to be a novel histone modifier participating in the indirect regulation of Ser activity.

Due to the limitation of knockdown assay, we could not reveal a long-term effect of suppression of MTA1 on testicular function. We tried to answer this question from a devious angle. The deteriorating effects of hyperthermia have been described in many male infertility-related diseases such as cryptorchidism, varicocele and chronic fever [52]. The influence of varicoceles on testicular function is variable, leaving it apparently unaltered in some cases, and causing partial or total arrest of spermatogenesis in others. It is suggested that an infertile man with varicocele might have testicular damage reflected by abnormal histologic patterns, such as maturation arrest, before becoming azoospermic [53]. Immunoblotting analysis revealed that the expression of MTA1 was decreased in the testes of varicocele patients compared with that in normal group. In line with our *in vivo* model, pachytene spermatocytes and Ser were two main cell types devoid of MTA1 immunolocalization. These data suggest that the decrease in MTA1 expression may be related to the pathogenesis of infertility in patients with varicocele.

DNA arrays have been employed to link gene expression to mechanisms of heat toxicity on murine testis. The overall pattern of gene expression was one of cellular shutdown. This is intuitively understandable, wherein an acute environmental insult causes cellular activity to cease and certain protective mechanisms are therefore guaranteed to initiate a defensive or reparative response [41]. To this end, further analysis of MTA1 function in the testes exposed to various exogenous thermal factors would help elucidate the underlying molecular mechanisms leading to male infertility. The fact that decrease of MTA1 could be detected even earlier than the beginning of the damage of testicular cells after heat stress also warrants its diagnostic potential for male infertility induced by hyperthermia.

## Materials and Methods

### Ethics Statement

The Ethics Committee for Animal Experiments of the Fourth Military Medical University approved all animal work (Permit number: 10001) and the experimental protocols strictly complied with the institutional guidelines and the criteria outlined in the “Guide for Care and Use of Laboratory Animals”. All surgery was performed under sodium pentobarbital anesthesia, and all efforts were made to minimize suffering. For human samples, the written informed consents have been obtained from all participants. The use of the human tissue in this study was approved by the Human Research Committee of the Fourth Military Medical University for Approval of Research Involving Human Subjects. The protocol employed strictly conformed to the standards set by *The 2008 Revised Declaration of Helsinki*.

### Tissue samples and cells

Adult male mice (C57BL/6) were obtained from the Animal Research Center of the Fourth Military Medical University, Xi'an (China) and maintained on a 12-hour light: 12-hour dark in a 20–25°C environment. They were allowed to acclimatize for at least 1 week before the experiment. To induce the transient heat stress, males were subjected to a single heat stress as described elsewhere with little modification [54]. Briefly, each animal was anesthetized using sodium pentobarbital (Sigma, 5 mg/100 g), and the lower third of the body (hind legs, tail, and scrotum) was submerged in a water bath of 43°C for 20 min. Animals were then dried and returned to their cages. Control animals, designated as 0 h, were anesthetized and left at room temperature. They were sacrificed at 8 h, 24 or 48 h after hyperthermal exposure. For biochemical analysis, tissues were stored at −80°C until use. For histological studies, murine testes were fixed in Bouin's solution for about 24 hours. After dehydration, the testes were embedded in paraffin and further processed into 5-μm-thick sections for morphological examination. To assess testicular degeneration after heat stress, hematoxylin-eosin (H&E) stained transverse testis sections were evaluated for the presence of ‘normal tubules’ if the general architecture showed normal spermatogenesis with all the layers of spermatogenic cells associated with each of the XII stages of spermatogenesis, or ‘degenerated’ if they showed depletion of all or some of germ cell layers usually associated with vacuolization within the seminiferous tubule. One hundred seminiferous tubules in a randomly selected section of the testis from five mice in each group were selected to quantify the relative percentage of degenerated tubules. This quantification was performed blindly and by two independent investigators.

Normal human testes were obtained from three victims of traffic accidents aged 25–40 years as described in previous publication [44]. Two biopsy specimens of histologically arrested testis (at the round spermatid level) were obtained from two patients, 29 and 37 years old, with varicocele at the male infertility clinic. Tissues were processed as described above.

Spermatogenic cell line GC-2spd (ts) was obtained from American Type Culture Collection (Rockville, MD) and maintained in Dulbecco's modified Eagle's medium (Gibco, Grand Island, NY) supplemented with 10% fetal calf serum (Life Technologies, Inc., CA), 1% nonessential amino acids solution (Gibco), and 100 lU/ml penicillin at 39°C in a humidified atmosphere of 10% CO_2_ in air. For temperature-shift experiments, cells were maintained at 32°C. After 6 h, cells were harvested and either fixed using 10% neutral buffered formalin for 10 minutes or stored at -80°C until use.

Testicular cells were prepared as previously reported [34]. Briefly, testes were decapsulated and digested for 15 min in 0.25% (w/v) collagenase (type IX, Sigma) at room temperature with constant shaking. Seminiferous tubules were then cut in pieces using a sterile blade and further digested in minimum essential medium containing 1 mg/ml trypsin for 30 min at 30 °C. Digestion was stopped by adding 10% fetal calf serum and the released germ cells were collected after sedimentation (10 min at room temperature) of tissue debris. Germ cells were centrifuged for 10 min at 1,500 rpm at 4°C and the pellet resuspended in 20 ml of elutriation medium (120.1 mM NaCl, 4.8 mM KCl, 25.2 mM NaHCO_3_, 1.2 mM KH_2_PO_4_, 1.2 mM MgSO_4_(7H_2_O), 1.3 mM CaCl_2_, 11 mM glucose, 1× essential amino acid (Life Technologies, Inc.), penicillin, streptomycin, 0.5% bovine serum albumin). Homogeneity of cell populations ranging between 80 and 85% (pachytene spermatocytes) and 95% (round spermatids) was routinely monitored morphologically. Primary pachytene spermatocytes were cultured in minimum essential medium (Gibco) and supplemented with 0.5% BSA (Sigma-Aldrich), 1 mM sodium pyruvate, and 2 mM lactate at 32°C in a humidified atmosphere containing 95% air and 5% CO_2_ as described previously [55]. Cells were treated overnight with 10 µM U0126 (EMD) before the addition of DMSO (Sangon Biotech Co., Ltd, Shanghai, China) or 0.5 µM OA (Invitrogen, Beijing, China) for 4–6 h to induce metaphase entry. For heat stress, primary cells were exposure at 43°C for 1 h and then cultured at 32.5°C for 23 h followed by harvest from 0 h to 2.5 h. For isolation of Ser after *in vivo* siRNA experiment, after being dispersed (but not fragmented) in 0.1% collagenase (type IV) and 0.04% DNase I (all from Sigma) in 1× Hanks fluid (pH 7.4) at 34°C for 10–15 min with constant shaking (100 oscillations/min), the seminiferous tubules were incubated in 1× Hanks fluid (pH 7.4) containing 0.04% DNase I, 0.05% hyaluronidase, and 0.5% trypsin for at least 10 min at 34°C with agitation. The fragmented tubules were allowed to settle and cells were subsequently centrifuged at 900 rpm and at 700 rpm (each for 3 min). Cells in the supernatant were collected and cultured (in DMEM/F12 medium containing 5% FCS) overnight. Sertoli cells attached to the bottom and acquired an irregular shape, whereas the germ cells did not attach and could easily be removed by repeated washing. The purity of isolated testicular cells could reach 85% (pachy) and 90% (Ser), respectively. Further confirmation of the cell types were carried out using RT-PCR analysis of specific cell markers as described in the following part.

### *In vitro* and *in vivo* siRNA treatment

*In vitro* siRNA treatment was performed as previous report [56]. In brief, GC-2spd (ts) was plated in six-well culture plates in DMEM media without antibiotics. The cells were allowed to grow to 50–60% confluence before being transfected with siRNA against *MTA1* (sc-35982, Santa Cruz Biotechnology, Santa Cruz, CA) or with a control siRNA (sc-37007, Santa Cruz Biotechnology, Santa Cruz, CA). Subsequent incubation of cells in transfection medium along with the transfection reagent was strictly followed the Santa protocol. The cells were incubated with the siRNA mixture for a period of 48 h before being subjected to either mRNA and protein analyses or heat stress treatment.

For *in vivo* siRNA experiment, adult mice were anesthetized as described above. Testes were pulled out from scrotum, and about 30 µl of plasmid DNA solution (sc-35982, Santa Cruz Biotechnology, Santa Cruz, CA) was injected into the rete testis using glass capillaries under a dissecting microscope as previously described [57]. Electric pulses were delivered using BTX ECM-830 Electro Square Porator (Waukegan, IL, USA). Square electric pulses were applied four times, and again four times in the reverse direction at 50 V for 50 ms for each pulse. The testes were then returned to the scrotum and skin was closed with sutures. The total RNA and protein of testis were collected for assessment 48 h after injection. All efforts were made during the whole process to minimize animal suffering.

### RT-PCR and QRT-PCR

Total RNA was extracted from cells or mouse testis using RNeasy Mini Kit (QIAGEN Inc., Valencia, CA, USA) according to the manufacturer's instructions. Routine DNase (Applied Biosystems/Ambion, Austin, TX, USA) treatment (1 U DNaseI per µg total RNA) was performed before reverse transcription. First-strand cDNA was synthesized using 1 µg RNA with Superscript III (Rnase H-Reverse Transcriptase; Invitrogen), according to the manufacturer's instructions and PCR was set up according to Promega's reverse transcription system protocol. The details of primers used in this study were listed in Table 2. The amplification of *18S* and *GAPDH* were served as internal controls. All PCR reactions for all samples were repeated at least three times. PCR products were then quantified by SYBR green intercalation using the MiniOpticon™ system (Bio-Rad Laboratories, Inc., Hercules, CA, USA). Standard curves were constructed for *MTA1* (specific target) and *18S* (internal control) by plotting values of CT (the cycle at which the fluorescence signal exceeds background) versus log cDNA input (in nanograms). Accordingly, CT values from each experimental sample were then used to calculate the amount of *MTA1* and *18S* mRNAs relative to the standard. For each sample, results in terms of *MTA1* expression levels were normalized to those of the internal control *18S*.

**Table 2:** Primer sequences for RT-PCR or real-time PCR of target genes.

Gene	GenBank access number	Primer sequence	product length (*bp*)
*18S*	M10098.1	F: CTCGCCGCGCTCTACCTACCTA	
		R: ATGAGCCATTCGCAGTTTCACTGTA	120
*MTA1*	AF288137.1	F: CAGTGTCGCCTCTGCGCATC	
		R: TCCACTGCTCCGAGCTGGAA	141
*Gapd-s*	M60978.1	F: CTGGCCAAGCCTGCTTCTTAC	
		R: CAAGGAGGGGCCTTTTGTGTTAC	286
*Proacrosin*	NM013455.3	F: TCCCCAAATACCCCACACCTG	
		R: CCCACCACTGTCCCCCTG	217
*Sprm-1*	NM029315.1	F: TGCGACCCCTGCTGAAAATG	
		R: GAGGCGCCCAGCAATATAGC	203
*cyclin A1*	NM007628.3	F: ATTGCAGCTTGTCGGGACAG	
		R: TGGTGGTTGGAACGGTCAGA	179
*GAPDH*	M32599.1	F: GGGTGAGGCCGGTGCTGAGT	
		R: TGACCCGTTTGGCTCCACCCT	98
*Vasa*	NM001145885.1	F: AGGAAGCAGAGATATTGGCGAGTCT	
		R: ACCTCTGTTTCCAAAGCCCTTTCCT	91
*pgk-2*	NM031190.2	F: AGGCCACCTCCAATGGCTGTGT	
		R: TGTGCGCAGGAAACAGGAAGCAA	210
*Gata-1*	NM_008089.1	F: GTGAACTGTGGAGCAACG	
		R: TTGACAGTTAGTGCATTGGG	174
*Prm-1*	NM_013637.4	F: ACTCCTGCGTGAGAATTTTAC	
		R: TTATTGACAGGTGGCATTGTT	113

Primers for CHIP assay and for Sertoli function analysis after *in vivo* RNAi were referenced at Literature 59 and 48, respectively.

### Western blot analysis

Protein samples were prepared in ice-cold RIPA buffer (Tris-HCl 50 mM, NaCl 150 mM, Triton X-100 1% vol/vol, sodium deoxycholate 1% wt/vol, and SDS 0.1% wt/vol pH 7.5) supplemented with complete proteinase-inhibitor cocktail tablets (Roche Diagnostic, Mannheim, Germany). Protein was separated on 8–15% SDS/PAGE and transferred to nitrocellulose membrane (Millipore, Bedford, MA, USA). Membranes were then incubated with primary antibodies including anti-MTA1 (Santa Cruz biotechnology, CA, USA; dilution 1∶500), anti-β-actin (Santa Cruz biotechnology, CA, USA; dilution 1∶2000), anti-p53 (Santa Cruz biotechnology, CA, USA; dilution 1∶1000), anti-acetyl-p53 (Millipore, MA, USA; dilution 1∶1000) and anti-p21 (Santa Cruz biotechnology, CA, USA; dilution 1∶1000) in blocking solution overnight at 4^o^C. Positive signals were finally detected by using an ECL kit (Amersham Biosciences, Buckinghamshire, UK).

### Immunofluorescence and immunohistochemistry

GC-2spd (ts) were fixed in 4% paraformaldehyde and washed three times with PBS. Cells were permeabilized with 0.1% Triton X-100 for 10 min and then incubated for 1 h in 0.5% BSA. Cells were washed three times with PBS and incubated overnight at 4°C with antibody against MTA1 (1∶200) followed by 1 h of incubation with FITC-labeled anti-goat IgG (dilution 1∶800; Sigma). Nuclear were visualize by 10-minute staining of DAPI (dilution 1∶2000; Sigma). Slides were finally analyzed by microscopy using an inverted microscope (Axio Imager M1 microscope, Zeiss).

Streptavidin-biotin complex (SABC) immunohistochemical method was conducted as previously described [17]. In brief, the sections were exposed to 0.3% hydrogen peroxide in methanol for 30 min to destroy endogenous peroxides activity after deparaffinization and rehydration. The slides were then incubated with the goat anti-MTA1 antibody (Santa Cruz Biotechnology, Santa Cruz, CA, USA; 1∶150 dilution) diluted in PBS, at 4°C overnight in a moist box. Biotinylated rabbit anti-goat IgG (1∶800 dilution; Sigma) was incubated on the sections for 1 h at room temperature and detected with streptavidinperoxidase complex. Peroxidases were detected with 0.7mg/ml 3-3′-diaminobenzidine tetrahydrochloride (Sigma, St. Louis, MO, USA) in 1.6mg/ml urea hydrogen peroxide. The sections were subsequently counterstained with hematoxylin for 40 sec. Control slides were incubated with a nonimmune serum instead of primary antibody. The immunohistochemical staining for MTA1 protein in mouse and human testes was evaluated by scanning the entire tissue specimen under low-power magnification (×100) and then confirmed under high-power magnification (×400) by two pathologists. 50 areas (×400) were randomly selected and totally 10 sections of each sample were included in the quantitative analysis. The signal intensity was stratified as strong staining (+++), moderate staining (++), weak staining (+), no staining (-). For semi-quantitative evaluation of positive cell number, at least 1000 cells in each cell type was counted and stratified as [-/+] <10%, [+] 10–25%, [++] 25–50%, [+++] 50–75% or [++++] >75% positive.

### In situ end-labeling of fragmented DNA

Apoptotic cells were identified by the TUNEL technique (in situ end-labeling of fragmented DNA), using In Situ Cell Death Detection Kit, POD. (Roche Applied Science, Mannheim, Germany) following instructions of the manufacturer, on fixed cells or paraffin-embedded tissue sections.

### Quantification of the apoptotic cells

An apoptosis ELISA kit (Roche Diagnostics, Mannheim, Germany) was used to quantitatively measure cytoplasmic histone-associated DNA fragments as previously reported [58].

### Chromatin immunoprecipitation

Germ cells were incubated with 1% formaldehyde in medium for the last 10 min of culture, washed in cold PBS and harvested. Nuclei were isolated by lysing cells in hypotonic buffer (5 mM Pipes pH 8.0, 85 mM KCl and NP-40 0.5%). Nuclei were then re-suspended, lysed in a buffer containing 1% SDS, 10 mM EDTA and 50 mM Tris/HCl pH 8.1 and sonicated with 8 pulses (1−, 90% Amplitude), clarified on ProteinA/agarose/salmon sperm DNA (Millipore) and used (100 µg of DNA/sample) for immunoprecipitation with 2 µg of MTA1, HDAC2 antibodies or control IgG (Santa Cruz Biotechnology, Santa Cruz, CA, USA). Primers used for ChIP were listed in Table 2 [59].

### Immunoprecipitation analysis

Immunoprecipitation analysis was performed as reported before [60]. In brief, cell lysates were obtained using RIPA buffer containing a complete proteinase-inhibitor cocktail tablets (Roche Diagnostic, Mannheim, Germany) and centrifugation at 5000 g at 4°C for 10 min. The lysates were incubated with mouse anti-p53 antibody at 4°C overnight. On the following day, protein G-Sepharose (Pierce, Rockford, IL, USA) was added into lystates and the compound was incubated at 4°C for another 2 h. Immunocomplexes were finally eluted from the sepharose beads by boiling in Laemmli sample buffer and subjected to SDS-PAGE of immunoblotting analysis with rabbit anti-HDAC2 antibody (Abcam, Shatin, N.T., Hong Kong, China).

### Statistical analysis

Experiments were repeated at least three times, and one representative from at least three similar results is presented. The significance of the results was determined by using the one-way ANOVA parametric test. Statistical differences were considered significant at P<0.05. Data were presented as the mean±SD.

## Supporting Information

Figure S1 **Identification of isolated primary pachytene spermatocytes by RT-PCR analysis.** *Gata-1, Prm-1* and *cyclin A1* were employed as specific markers for Ser, Rsd and Pachy, respectively.
(TIF)

Figure S2 **Isolated pachytene spermatocytes were stimulated to enter meiotic divisions by treatment of OA.** Control (DMSO) or treated (OA) spermatocytes were analyzed by immunoblotting (**A**) or immunofluorescence (**B**) assays with the anti-phosphoH3 (p-H3) antibody, *Bar = 10 µm*. Appearance of phosphorylated H3 indicates that spermatocytes have progressed to the M phase of the first meiotic division. **C** Upreguation of phospho-Erks was only detected by western blot in primary spermatocytes treated with OA.
(TIF)

Figure S3 ***MTA1/18S*** **relative expression level in primary spermatocytes at different time-points after exposure to heat stress was examined by real-time PCR.** Data are expressed as mean±SEM (n = 3; ^*^p<0.05, ^**^ p<0.01 vs. 0 h).
(TIF)

Figure S4 **Knockdown of** ***MTA1*** **expression in GC-2spd (ts) by RNAi. A RT-PCR analysis of** ***MTA1*** **expression after** ***in vitro*** **RNAi.** *18S* was served as an internal control. **B** PCR products were then quantified by SYBR green intercalation in real-time PCR. Data are expressed as mean±SEM (n = 3; ^*^p<0.05 vs. control). **C** Western analysis of MTA1 protein in GC-2spd (ts) after RNAi treatment. β-actin was used to confirm equal loading. Three separate experiments were repeated and one representative result was presented. **D** Dose-dependent interference effect of siRNA against *MTA1* was revealed by western blot analysis. CT, group treated with control siRNA; KD, group treated with *MTA1* siRNA.
(TIF)

Figure S5 **Effect of** ***in vivo*** **RNAi on the expression of** ***MTA1*** **was further confirmed by RT-PCR analysis on isolated Ser and pachy from siRNA treated testes.** **A** Identification of isolated Ser and pachy by RT-PCR analysis. **B** Transcriptional expression of *MTA1* in isolated Ser and pachy.
(TIF)

Figure S6 **48 h after** ***in vivo*** **knockdown of MTA1, the mouse testis was subjected to transient heat stress as described in** ***Materials and methods*** **.** Changes in testicular expression of functional genes of Ser were then evaluated using real-time PCR. Data are expressed as mean±SEM (n = 3; ^*^p<0.05 vs. control).
(TIF)

Figure S7 **MTA1 expression was impaired in the testis of a mouse transient scrotal heat stress model.** **A** Murine testicular sections collected at 8 h (n = 5) or 24 h (n = 5) after hyperthermia exposure were subjected to TUNEL staining. Apoptotic activity was quantified as the number of cells positive for TUNEL staining within 50 seminiferous tubules. ^#^p>0.05 or ^**^p<0.01 vs. 0 h group. **B** Effect of heat stresses on the expression of *MTA1*, *pgk-2*, *vasa* and *GAPDH* at different time-points was elucidated at the transcriptional level. C, control group; H, hyperthermia-treated group. **C** Immunolocalization of MTA1 in the testicular sections at 0 h and 8 h after hyperthermia exposure. Replacement of the primary antibody with normal goat IgG was served as negative control (NC). Arrows indicate pachytene spermatocytes. *Bar = 25* *µm*.
(TIF)
