# Supplementary figures and images for: Transient Protection from Heat-Stress Induced Apoptotic Stimulation by Metastasis-Associated Protein 1 in Pachytene Spermatocytes
Source: PLoS One. 2011 Oct 12;6(10):e26013. doi: 10.1371/journal.pone.0026013 (PMC3192157; doi:10.1371/journal.pone.0026013)

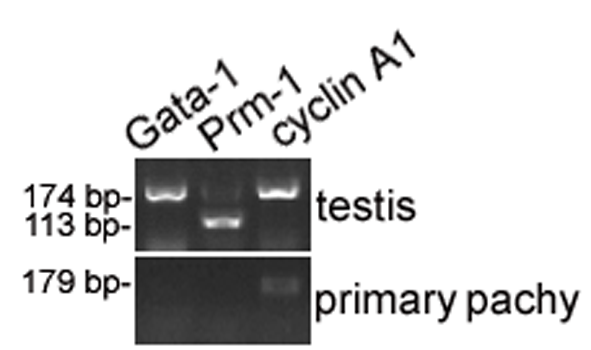

Supplement: Figure S1 — Identification of isolated primary pachytene spermatocytes by RT-PCR analysis. Gata-1, Prm-1 and cyclin A1 were employed as specific markers for Ser, Rsd and Pachy, respectively. (TIF) [file pone.0026013.s001.tif]

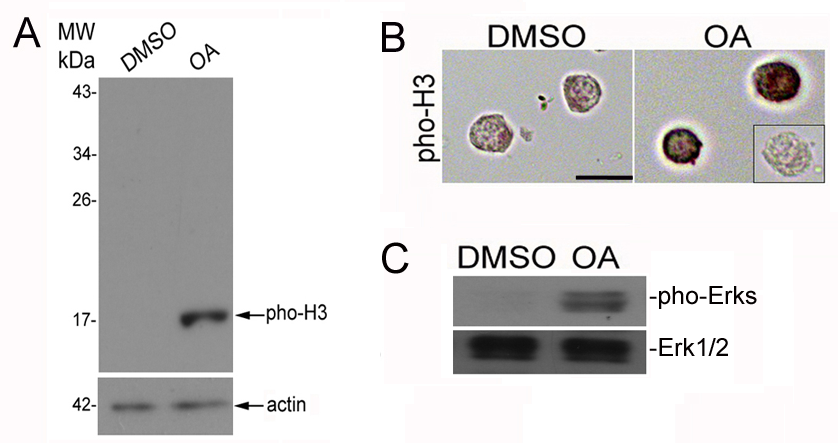

Supplement: Figure S2 — Isolated pachytene spermatocytes were stimulated to enter meiotic divisions by treatment of OA. Control (DMSO) or treated (OA) spermatocytes were analyzed by immunoblotting (A) or immunofluorescence (B) assays with the anti-phosphoH3 (p-H3) antibody, Bar = 10 µm. Appearance of phosphorylated H3 indicates that spermatocytes have progressed to the M phase of the first meiotic division. C Upreguation of phospho-Erks was only detected by western blot in primary spermatocytes treated with OA. (TIF) [file pone.0026013.s002.tif]

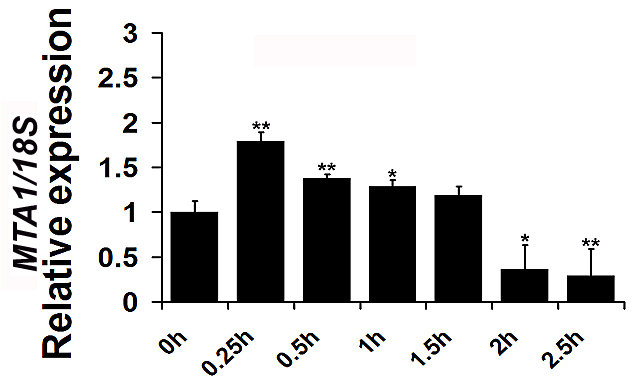

Supplement: Figure S3 — MTA1/18S relative expression level in primary spermatocytes at different time-points after exposure to heat stress was examined by real-time PCR. Data are expressed as mean±SEM (n = 3; *p<0.05, ** p<0.01 vs. 0 h). (TIF) [file pone.0026013.s003.tif]

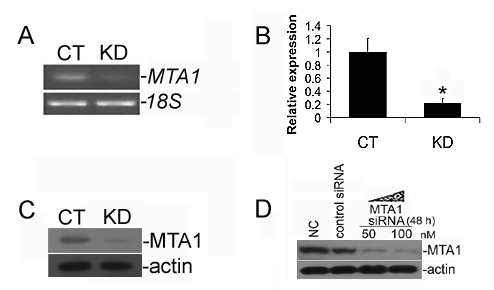

Supplement: Figure S4 — Knockdown of MTA1 expression in GC-2spd (ts) by RNAi. A RT-PCR analysis of MTA1 expression after in vitro RNAi. 18S was served as an internal control. B PCR products were then quantified by SYBR green intercalation in real-time PCR. Data are expressed as mean±SEM (n = 3; *p<0.05 vs. control). C Western analysis of MTA1 protein in GC-2spd (ts) after RNAi treatment. β-actin was used to confirm equal loading. Three separate experiments were repeated and one representative result was presented. D Dose-dependent interference effect of siRNA against MTA1 was revealed by western blot analysis. CT, group treated with control siRNA; KD, group treated with MTA1 siRNA. (TIF) [file pone.0026013.s004.tif]

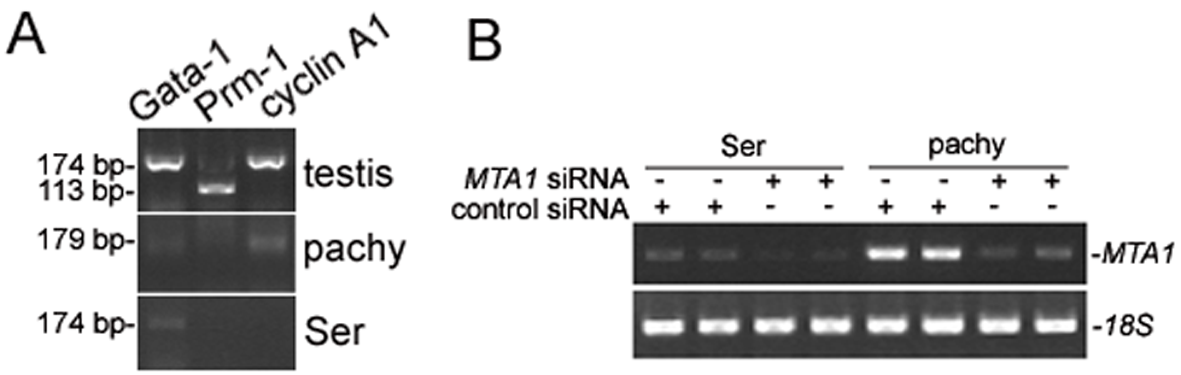

Supplement: Figure S5 — Effect of in vivo RNAi on the expression of MTA1 was further confirmed by RT-PCR analysis on isolated Ser and pachy from siRNA treated testes. A Identification of isolated Ser and pachy by RT-PCR analysis. B Transcriptional expression of MTA1 in isolated Ser and pachy. (TIF) [file pone.0026013.s005.tif]

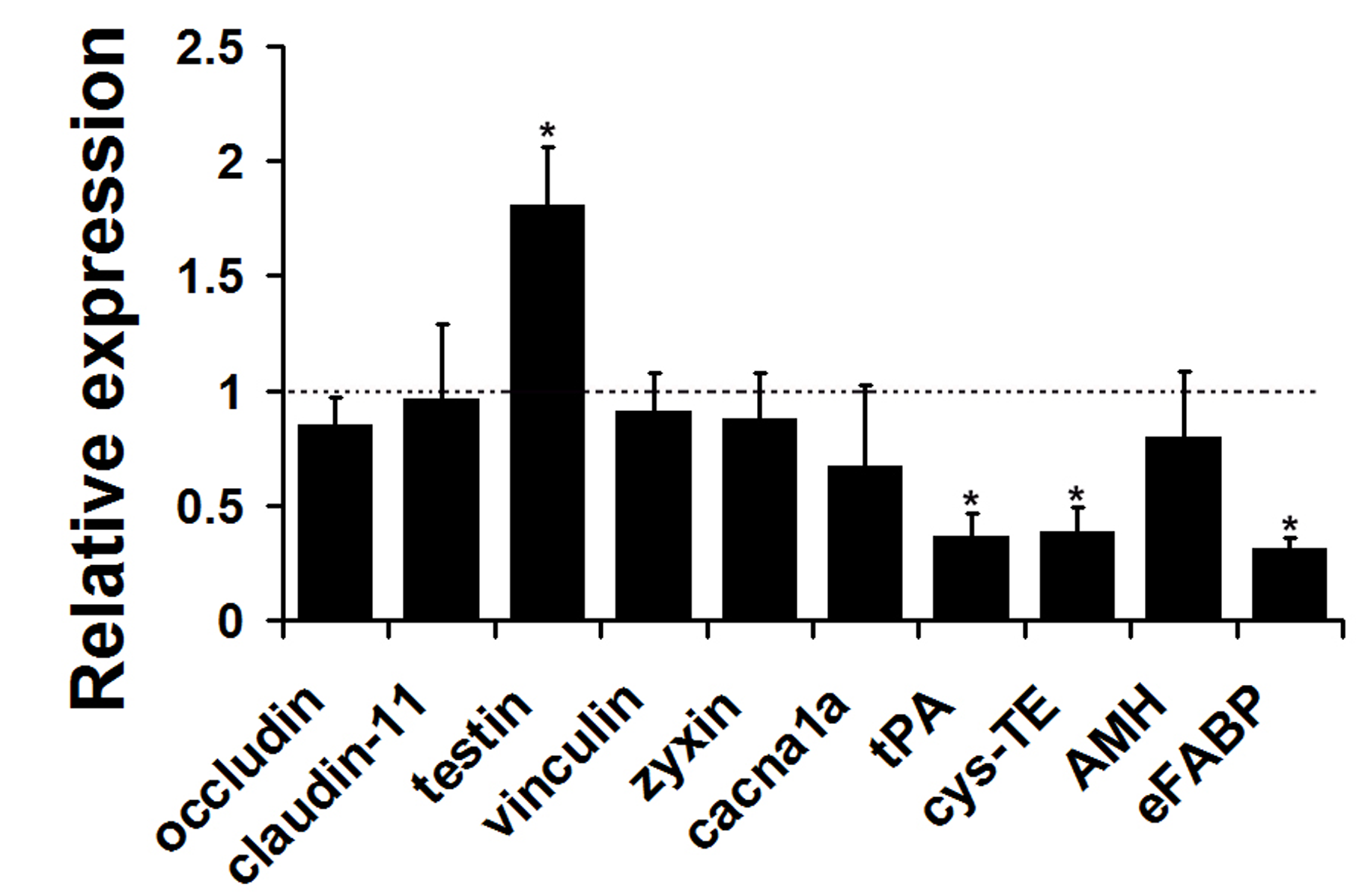

Supplement: Figure S6 — 48 h after in vivo knockdown of MTA1, the mouse testis was subjected to transient heat stress as described in Materials and methods . Changes in testicular expression of functional genes of Ser were then evaluated using real-time PCR. Data are expressed as mean±SEM (n = 3; *p<0.05 vs. control). (TIF) [file pone.0026013.s006.tif]

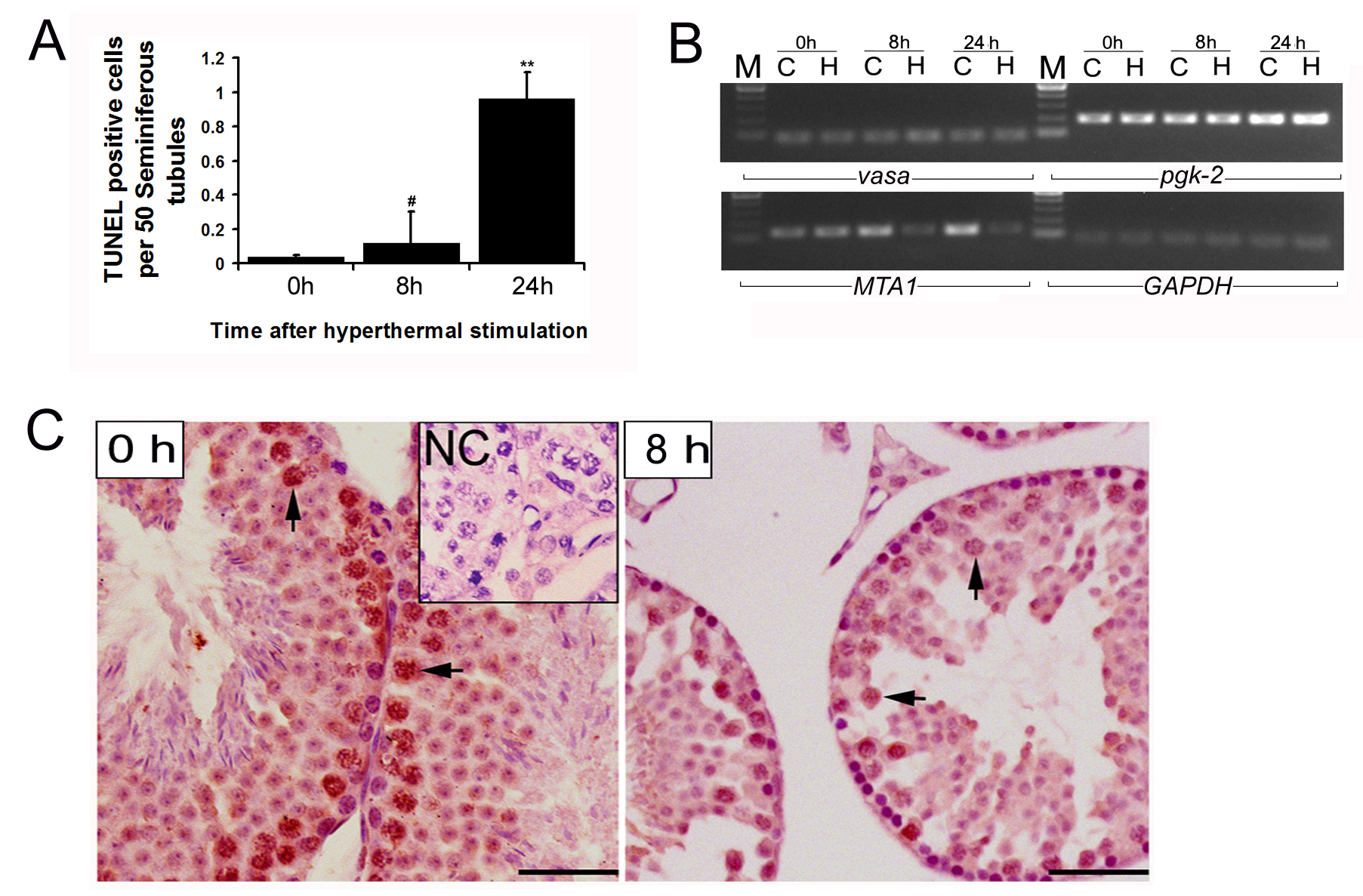

Supplement: Figure S7 — MTA1 expression was impaired in the testis of a mouse transient scrotal heat stress model. A Murine testicular sections collected at 8 h (n = 5) or 24 h (n = 5) after hyperthermia exposure were subjected to TUNEL staining. Apoptotic activity was quantified as the number of cells positive for TUNEL staining within 50 seminiferous tubules. #p>0.05 or **p<0.01 vs. 0 h group. B Effect of heat stresses on the expression of MTA1, pgk-2, vasa and GAPDH at different time-points was elucidated at the transcriptional level. C, control group; H, hyperthermia-treated group. C Immunolocalization of MTA1 in the testicular sections at 0 h and 8 h after hyperthermia exposure. Replacement of the primary antibody with normal goat IgG was served as negative control (NC). Arrows indicate pachytene spermatocytes. Bar = 25 µm. (TIF) [file pone.0026013.s007.tif]
